# Study on the correlation between the circle of Willis structure and collateral circulation in bilateral carotid artery occlusion

**DOI:** 10.1007/s10072-021-05265-4

**Published:** 2021-04-23

**Authors:** Yang Liu, Lei Zhu, Bei Hou, Tuoyi Wang, Dechao Xu, Chenxi Tan, Huiyi Zhang, Chunyan Li, Jianan Wang

**Affiliations:** 1 Department of Neurology, The Second Affiliated Hospital of Qiqihar Medical university, No.37 West Zhonghua Road, Qiqihar, 161006 Heilongjiang China; 2 Department of Nucleus Radiation-related Injury Treatment, PLA Rocket Force Characteristic Medical Center, No.16 Xinjiekouwai Street, Xicheng District, Beijing, 100088 China; 3 Department of Urology, PLA Rocket Force Characteristic Medical Center, No.16 Xinjiekouwai Street, Xicheng District, Beijing, 100088 China; 4 College of Food and Biological Engineering, The Qiqihar University, No.42 Wenhua Street, Qiqihar, 161006 Heilongjiang China; 5 Center of Pathology, The Qiqihar Medical university, No.333 North Bukui Road, Qiqihar, 161006 Heilongjiang China

**Keywords:** Circle of Willis, Carotid stenosis/occlusion, Collateral circulation, Blood stealing

## Abstract

**Background and purpose:**

Bilateral carotid artery occlusion (CAO) is a rare condition and the collateral circulation is more complicated than in unilateral CAO. The circle of Willis (CoW) is the most important collateral circulation compensation pathway in CAO. However, the specific role of CoW in the collateral circulation compensation pathway of CAO has not been fully elucidated. The purpose of this study is to investigate the role of CoW in the collateral circulation compensation pathway of CAO.

**Materials and methods:**

Clinical, imaging, and hemodynamic data of 30 patients with bilateral CAO were collected to analyze the collateral blood flow compensation pathway and its characteristics, and to examine the correlation between the structure of the CoW and the collateral circulation of bilateral CAO.

**Results:**

This paper summarized 30 patients with bilateral CAO. There were 0 cases of the CoW complete type, 18 cases of the partially complete type (60%), and 12 cases of the incomplete type (40%). For the partially complete type cases, there were 14 complete anterior circulation cases (46.7%). The collateral circulation collateral circulation pathway included 14 cases with anterior communicating artery(ACoA), 7 cases with posterior cerebral artery (PCA)-middle cerebral artery (MCA) leptomeningeal anastomosis (LMA), 5 cases with ophthalmic artery(OA), 3 cases with lateral posterior communicating artery(PCoA), 1 case with internal carotid artery (ICA) stealing, 1 case with new Moyamoya vessels, and 4 cases of other types. There were four cases (13.3%) with complete posterior circulation, including four cases with bilateral PCoA, three cases with PCA-MCA LMA, and two cases with OA. There were 12 cases (40%) with incomplete CoW, including 8 cases with PCA-MCA LMA, 3 cases with lateral PCoA, 1 case with anterior cerebral artery (ACA)-MCA LMA, 4 cases with OA, and 1 other case.

**Conclusion:**

The collateral circulation pathway differs among patients with different CoW structure types. When the CoW is partially complete, it mainly provides blood flow compensation to the ischemic area through primary collateral circulation. When the CoW is incomplete, it mainly provides blood flow compensation to the ischemic area through secondary collateral circulation.

## Introduction

Carotid artery stenosis is an important cause of ischemic stroke, and the incidence of carotid artery stenosis in patients with ischemic stroke is 15–20% [1]. In the case of CAO (severe stenosis/occlusion), the establishment of collateral circulation can indirectly supply the stenosis/occlusion of the cerebral artery blood supply area, increase cerebral blood flow to maintain adequate oxygenation and cell function in the ischemic area, and reduce the infarct volume [2]. The CoW is the most important collateral circulation compensation pathway in the cranium. It connects the left and right hemispheres of the brain with anterior and posterior circulation. Studies have shown that the collateral circulation capacity of the CoW is closely related to its structural integrity. The complete CoW can alleviate ischemia-reperfusion injury and save the ischemic penumbra, thus improving the prognosis of patients with cerebral infarction, by decompensating the anterior and posterior arteries primary collateral arteries [3]. Compared with unilateral carotid stenosis, bilateral carotid stenosis has a lower incidence and collateral compensation is more complex [4], so there are few studies at present.

In this study, clinical, imaging, and hemodynamic data of patients with bilateral CAO were retrospectively analyzed to explore the correlation between the structure of the CoW and collateral circulation of patients with bilateral CAO in order to provide a scientific basis for clinicians to better analyze the condition and prognosis of patients with complex CAO.

## Materials and methods

### Study subjects

This study included patients treated in the outpatient or inpatient department of the Second Affiliated Hospital of Qiqihar Medical College or the PLA Rocket Force Characteristic Medical Center from May 2017 to May 2020. All patients were chronic asymptomatic. Thirty patients with bilateral CAO (including common carotid artery [CCA], ICA, and external carotid artery [ECA] severe stenosis/occlusion) were confirmed by carotid ultrasound, transcranial Doppler ultrasound (TCD), head and neck CT angiography (CTA), or digital subtraction angiography (DSA). Among them, there were 24 males and 6 females, aged 48 to 82 years, with an average age of 64 years. Inclusion criteria were the following: All the patients in the group met the evaluation criteria of severe carotid artery stenosis or occlusion in the North American Symptomatic Carotid artery Endarterectomy Trial (NASCET) collaboration group. The standard of severe stenosis is 70–99% stenosis. Patients were enrolled with or without symptoms of ischemic stroke or transient ischemic attack in the carotid region. Patients with mild or moderate bilateral carotid artery stenosis or incomplete vascular ultrasound, imaging, or clinical data were excluded.

### Methods

The disease assessment team was composed of three physicians, including two associate chief physicians of neurology and one associate chief physician of the imaging department. In this study, the film evaluation was blind. TCD, CTA, and DSA were combined to evaluate the carotid artery lesion, CoW, and collateral circulation pathways. The structure of the CoW and collateral cycle was mainly evaluated, and the compensatory capacity of the collateral cycle was not evaluated.

#### Collateral circulation assessment methods

Vivid E9 color ultrasonography and a Sonara transcranial and peripheral vascular Doppler diagnostic system were used in this study. By accurately measuring cerebral artery vessel diameter and comparing the blood flow velocity, blood flow direction, and neck pressure, the lateral branches of ACoA, PCoA, OA, LMA, and ICA stealing and other lateral branches were distinguished. Collateral flow through the anterior communicating artery, posterior communicating artery, ophthalmic artery, and leptomeningeal arteries could be directly or indirectly detected by TCD. The sensitivities of TCD in detecting a patent anterior communicating artery and collateral flow through the basilar artery were reported to be 95% and 87%, and the specificities were 100% and 95% [5]. The sensitivity and specificity of flow diversion by TCD for predicting the presence of leptomeningeal collateral flow in DSA were, respectively, 81.1% and 76.7%, and the positive and negative predictive values were, respectively, 70.8% and 85.2% in a previous report [6]. Using a Toshiba Aquillon series 64-slice spiral CT machine, the flat scan and enhanced scan sequences were designed using vascular subtraction technology. The subtraction and vascular reconstruction of the images were carried out by combining the maximum density projection, multiplane reconstruction, volume reconstruction, and surface reconstruction to evaluate the collateral circulation pathway. CTA is also a non-invasive method that bears a high accuracy in assessing the patency of the arterial segments in the circle of Willis, with >90% agreement with DSA, but its sensitivity (53%) is limited in depicting hypoplastic arterial segments [7]. To find all kinds of collateral blood flow dynamic compensation pathways, DSA examination was performed using the Philips FD20 digital subtraction angiography system, femoral artery puncture was performed using a modified Seldinger technique, aortic arch angiography was performed using a Pigtail catheter, and selective cerebral angiography was performed on the subclavian artery and CCA on both sides by a single curved catheter.

#### Structure typing of the CoW

Previous studies have classified the CoW into two categories: complete and partially complete [8]. Recent studies have shown that Willis rings can be divided into complete and incomplete types according to their functions [9]. Based on these studies, we divide Willis rings into three types: complete, partially complete, and incomplete. A complete CoW means that each constituent vessel of the CoW is displayed continuously, and the starting and ending points of each vessel can be observed. A partially complete CoW is one in which the front and back cycles are partially incomplete. An incomplete CoW refers to the incomplete anterior and posterior loops—that is, at least one constituent vessel of the anterior and posterior circulation does not exist.

## Results

### Pathological vascular condition in patients with bilateral CAO

There were 21 cases of bilateral ICA occlusion (ICAO), including 6 cases of ipsilateral CCA occlusion (CCAO) with ICAO, 2 cases of ipsilateral ICAO with ECA occlusion (ECAO), and 1 case of ipsilateral CCAO and ICAO with ECAO.

### Features of collateral compensation in bilateral CAO patients with different types of the CoW

**Fig. 1:**
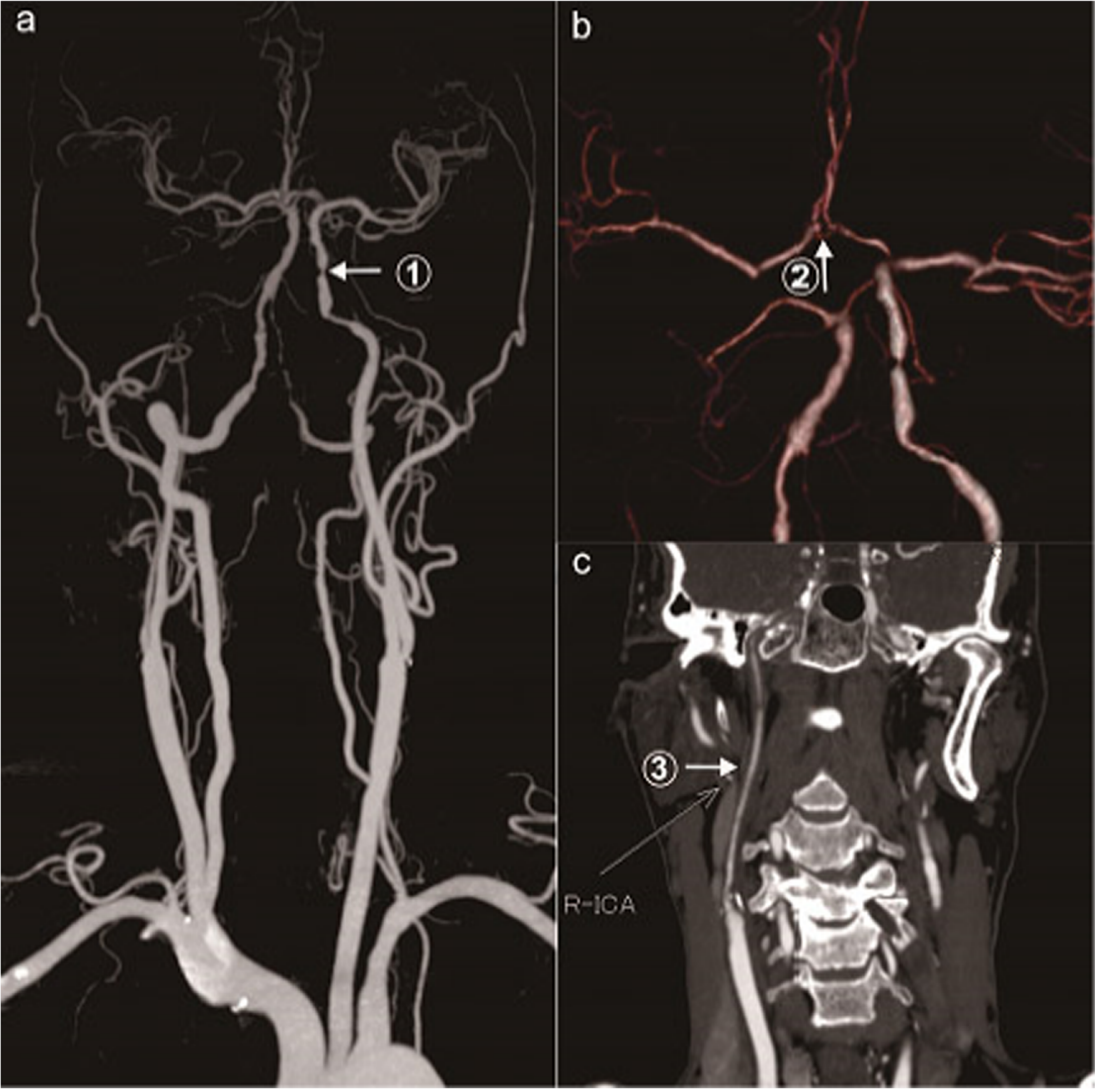
Complete anterior circulation of the CoW (case 26). **a** Arrow 1 shows severe stenosis in the C3 segment of the left ICA. **b** Arrow 2 shows the ACoA. **c** Arrow 3 shows severe stenosis/subocclusion of the right ICA C1-7

**Fig. 2:**
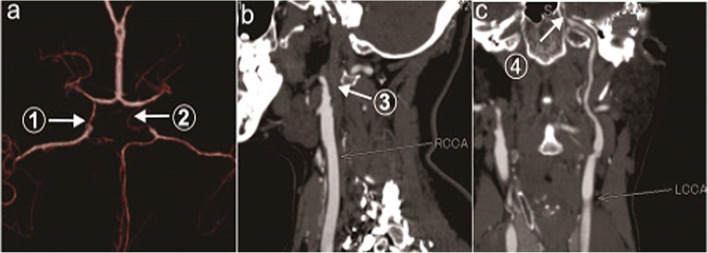
Complete posterior circulation of the CoW (case 17). **a** Arrows 1 and 2 show bilateral PCoA. **b** Arrow 3 shows the occlusion of the initial segment of the right ICA. **c** Arrow 4 shows severe stenosis at the C3 section of the left ICA

**Fig. 3:**
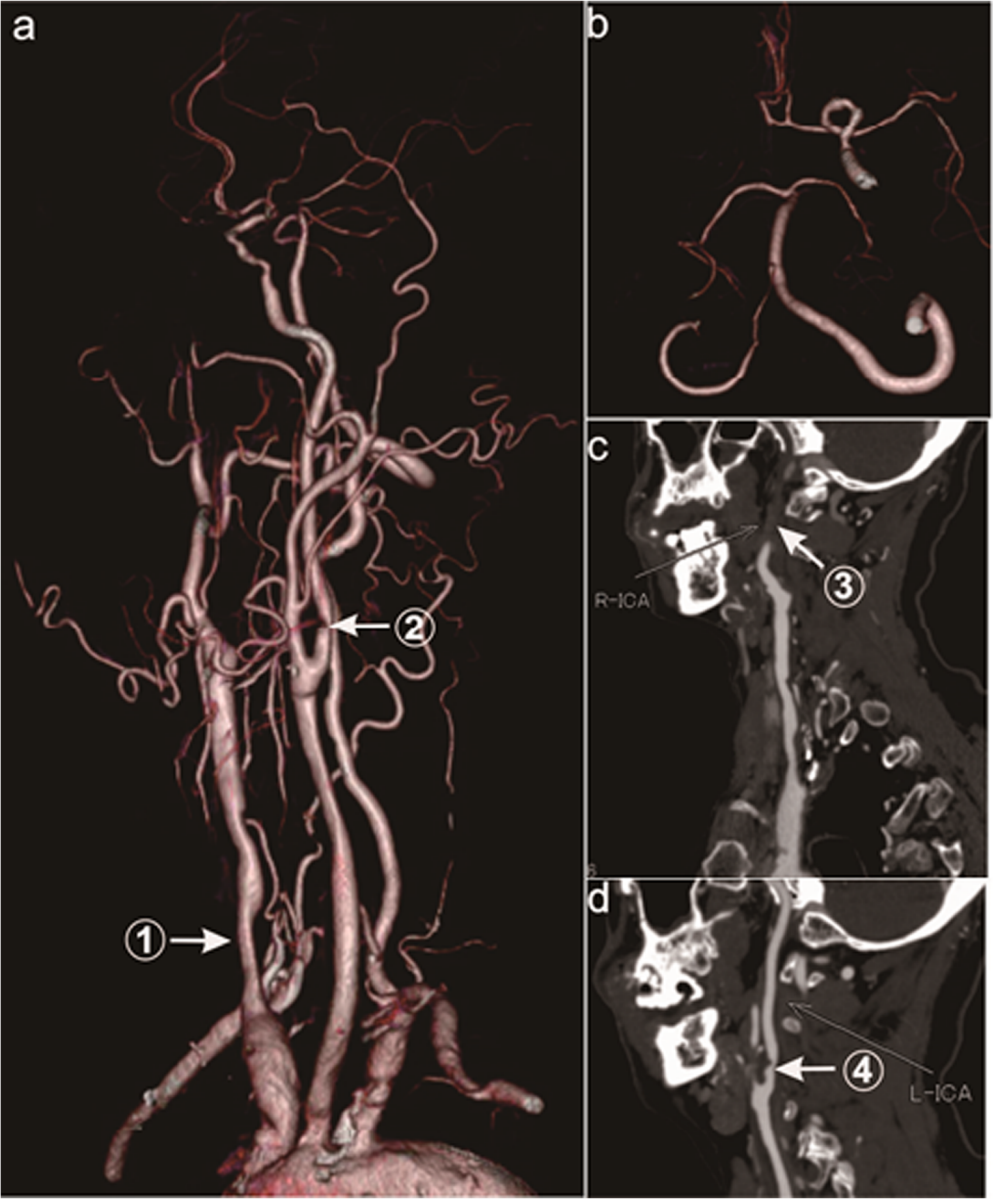
Incomplete type of the CoW (case 15). **a** Arrow 1 shows severe stenosis of the right CCA, and arrow 2 shows severe stenosis of the C1 section of the left ICA. **b** Bilateral ACA starts from the section of the left ACA without the PCoA. **c** Arrow 3 shows occlusion at the C1 segment of the right ICA. d Arrow 4 shows severe stenosis at C1 of the left ICA

There were zero cases of the CoW complete type. There were 18 cases (60%) of the CoW partially complete type, among them former circulation complete type 14 cases (46.7%). For the former circulation complete type, the collateral circulation included 14 cases with ACoA, 7 cases with PCA-MCA LMA , 5 cases with OA , 3 cases with lateral PCoA, 1 case with ICA stealing, 1 case with new Moyamoya vessels, and 4 cases of other types (Table 1, Fig. 1). For the posterior circulation complete type 4 cases (13.3%), the collateral circulation collateral circulation included 4 cases with bilateral PCoA, 3 cases with PCA-MCA LMA, and 2 cases with OA (Table 1, Fig. 2). There were 12 cases (40%) of the CoW incomplete type, and the collateral circulation collateral circulation included 8 cases with PCA-MCA LMA, 3 cases with lateral PCoA, 1 case with ACA-MCA LMA, 4 cases with OA, and 1 other case (Table 1, Fig.3).

**Table 1:** Collateral compensation characteristics of patients with bilateral CAO with different types of CoW (cases)

CoW type		Collateral circulation pathway							
		ACoA	PCoA	PCA-MCA LMA	ACA-MCA LMA	OA	ICA stealing	New vessels	Other
Complete		0	0	0	0	0	0	0	0
Partially complete (60%)	Anterior circulation complete (46.7%)	14	3	7	0	5	1	1	4
	Posterior circulation complete (13.3%)	0	4	3	0	2	0	0	0
Incomplete (40%)		0	3	8	1	4	0	0	1

*ACoA* anterior communicating artery, *PCoA* posterior communicating artery, *PCA* posterior cerebral artery, *MCA* middle cerebral artery, *ACA* anterior cerebral artery, *OA* ophthalmic artery, *ICA* internal carotid artery, *LMA* leptomeningeal anastomosis

## Discussion

Carotid artery stenosis is an important factor for inducing ischemic stroke in patients. Unilateral carotid artery stenosis is more common than bilateral carotid artery stenosis. A previous study reported that, among 27 bilateral patients, 11 patients were treated with carotid endarterectomy and 7 patients were treated with carotid artery stenting, and the incidence of main adverse events for the bilateral patients was 7.4% [10]. The incidence of CCA occlusion in stroke patients is estimated to vary from 1 to 5%, but the rate of recurrent stroke in bilateral ICA occlusion patients is about 10–17.4% [11]. It can be seen that bilateral CAO is rare in patients, but once the lesion occurs, the bilateral anterior circulation of cerebral artery will be in an ischemia state, which is prone to serious ischemic stroke events. Therefore, more attention should be paid to the characteristics of vascular stenosis and collateral circulation in patients with bilateral CAO. In this study, bilateral ICAO had the highest incidence in bilateral CAO with 21 cases (70%), among patients with bilateral carotid artery occlusion. The reason may be that the ICA has unique anatomic characteristics compared with the CCA and ECA. In particular, segment C1 of the ICA is more prone to forming atherosclerotic plaques and vascular stenosis because it is located in the bifurcation part of the vessel [12]. In this study, the disease incidence rate of segment C1 of the ICA was 66.7%. CCAO combined with ICAO had the second-highest rate (6 case), and the incidence of CCAO and ICAO combined with ECAO was the lowest (1 case).

Previous studies on bilateral severe carotid artery stenosis/occlusion mainly focused on the analysis of the therapeutic effect of carotid endarterectomy and carotid artery stent placement [13–15]. Few studies have performed collateral circulation assessment of bilateral severe carotid artery stenosis/occlusion, and its hemodynamic characteristics are still unclear. There is no effective basis for clinical analysis of complex cases of severe bilateral carotid artery stenosis/occlusion. Collateral circulation plays an important role in blood flow compensation for cerebral artery stenosis [16]. Studies have shown that in patients with severe carotid stenosis, the CoW plays a key role in the development of collateral flow [17, 18], which is crucial for ensuring cerebral ischemia-reperfusion. The CoW is a circular artery located at the base of the brain. It is composed of the A1 segment of the bilateral ACA, terminal of bilateral ICA, ACoA, P1 segment of the bilateral PCA, and PCoA [19]. It connects the anterior and posterior circulation with the left and right anterior circulation. When the vessels are narrowed or occluded, the ischemic sites can be compensated by the artery on the CoW. In the case of bilateral CAO, due to hemodynamic reasons such as the degree of cerebral artery stenosis and different perfusion pressure, cerebral artery blood flow can supply the low-pressure side from the high-pressure side through the ACoA and PCoA. Thus, the hypoperfusion caused by anterior circulation ischemia can be improved. In the general population, only 42–52% have a complete CoW structure [20]. It can be seen that the presence of a well-developed CoW is particularly important for the collateral circulation compensation of the bilateral CAO.

The integrity of the CoW is an important anatomical basis for the establishment of anterior and posterior collateral arteries. The more complete the structure of CoW, the greater the probability of the establishment of primary collateral circulation and the greater the chance of reperfusion of the ischemic penumbra [21]. The incidence of complete CoW is higher in women than in men [22]. An analysis of the CoW in 525 healthy people showed that only 20.9% had complete CoW. Among them, the vascular integrity of the anterior half of the CoW accounted for 80.95%, and the vascular integrity of the posterior half accounted for 20.95% [23]. Liu analyzed 139 patients with severe unilateral internal carotid artery stenosis/occlusion and found that only 18% of the CoW was complete [24]. In this study, there were only 6 females (20%) among 30 patients with bilateral CAO and zero patients with complete CoW. The reason for the lack of complete CoW may be that fewer cases were enrolled in this study, fewer women were enrolled in the study, and the variation rate of CoW in patients with CAO was higher. Meanwhile, there were 18 cases (60%) of partially complete CoW. In addition, there were 14 cases (46.7%) of complete anterior circulation but only 4 cases (13.3%) of complete posterior circulation, which was consistent with previous research results. It can be assumed that, compared with the complete CoW, the incidence of incomplete CoW in patients with ischemic cerebrovascular disease is higher.

The cerebral hemodynamic characteristics and perfusion pattern of patients with bilateral CAO were different from those of patients with unilateral CAO, and the cerebral blood flow self-regulation function was seriously impaired, so the hemodynamic disorder was more serious. Studies have reported that in the case of unilateral carotid artery stenosis, the ACoA collateral circulation is mainly started, and the contralateral carotid artery compensates for reduced cerebral perfusion on the affected side. Patients with bilateral carotid artery stenosis mainly rely on the PCoA collateral circulation for compensation, and the blood in the posterior circulation is supplied through the PCoA to the anterior circulation. When bilateral CAO occurs, due to the pressure difference in bilateral anterior circulation, the side with relatively mild stenosis can provide blood flow compensation to the side with relatively severe stenosis through the collateral branch of the ACoA [25**,** 26]. Other studies found that the incidence of posterior cerebral artery to middle cerebral artery LMA collateral circulation increased when bilateral CAO occurred. In the cases with intact anterior circulation of CoW in this study, the most common collateral collateral circulation pathway of the complete anterior CoW was the lateral branch of the ACoA (14 cases). The most common collateral circulation pathway in the cases with complete posterior circulation of CoW was the bilateral PCoA (4 cases). It can be seen that primary collateral compensation of the CoW still plays a major role in bilateral CAO with partially complete CoW. Meanwhile, in the CoW incomplete type patients, because the anterior and posterior circulation are incomplete, the LMA collateral circulation, OA, and other collateral circulation pathways (pericallosal artery anastomotic collateral circulation, dural artery-LMA collateral circulation, ICA stealing, etc.) were fully activated to supplement the insufficiency of primary collateral compensation, and the collateral circulation pathways were more complex and diverse. One of the most initiated collateral circulation pathways was PCA-MCA LMA collateral circulation with eight patients (66.7%). Therefore, in the case of bilateral carotid occlusion with incomplete CoW, the secondary collateral of the PCA-MCA LMA collateral plays a major compensatory role.

In summary, through retrospective analysis of imaging and hemodynamic data of 30 patients with bilateral CAO, the present study found that there were differences in collateral circulation among patients with different CoW types. The integrity of the CoW structure determined the establishment of collateral circulation in patients with bilateral CAO. When the CoW was partially complete, it mainly provided blood flow compensation to the ischemic area through the primary collateral circulation. When the CoW was incomplete, the primary collateral circulation could not be established, and the secondary collateral mainly provided blood flow compensation to the ischemic area.

## Conclusion

The collateral circulation pathway is different among patients with different CoW structure types. When the CoW is partly complete, it mainly provides blood flow compensation to the ischemic area through primary collateral circulation. When the CoW is incomplete, it mainly provides blood flow compensation to the ischemic area through secondary collateral circulation.

## Limitations

Since we enrolled chronic asymptomatic cases, only 30 patients were collected and no study was conducted on the complete CoW. We discussed the condition of collateral circulation open establishment without analyzing the compensatory capacity. We will carry out further research in these aspects in the future.

## Abbreviations

CAO: carotid artery occlusion
CCA: common carotid artery
CCAO: common CAO
CTA: computed tomography angiography
DSA: digital subtraction angiography
ECA: external carotid artery
ICA: internal carotid artery
MRA: magnetic resonance angiography
TCD: transcranial Doppler
ACoA: anterior communicating artery
PCoA: posterior communicating artery
PCA: posterior cerebral artery
MCA: middle cerebral artery
ACA: anterior cerebral artery
OA: ophthalmic artery
ICA: internal carotid artery
LMA: leptomeningeal anastomosis
